# Matrix-independent screening of defluorination *in vitro* and *in vivo*

**DOI:** 10.1128/mbio.01798-25

**Published:** 2025-08-18

**Authors:** Anitha T. Simon, Anthony G. Dodge, Julie Bondy, Madeline R. O’Connor, Alptekin Aksan, Lawrence P. Wackett

**Affiliations:** 1 Department of Mechanical Engineering, University of Minnesota Twin Cities 5635, Minneapolis, Minnesota, USA; 2 Biotechnology Institute, University of Minnesota Twin Cities 5635, Saint Paul, Minnesota, USA; 3 Department of Biochemistry, Molecular Biology and Biophysics, University of Minnesota Twin Cities 5635, Saint Paul, Minnesota, USA; 4 Program in Microbial Engineering, University of Minnesota Twin Cities 5635, Saint Paul, Minnesota, USA; Georgia Institute of Technology, Atlanta, Georgia, USA

**Keywords:** PFAS, pH indicator, screening, rapid method, defluorination, enzyme, bacteria, liquid media, agar plates, hydrogel

## Abstract

There is intense interest in biodegrading fluorinated pesticides and other commercial products, some of which are per- and poly-fluorinated alkyl substances, or PFAS. Enzymatic carbon-fluorine bond cleavage via hydrolytic, reductive, and eliminative mechanisms generates an organic product, fluoride anion, and a proton. Biodegradation is typically determined by tracking the organic product using liquid chromatography-mass spectrometry (LC-MS) or the anion determined by a fluoride-specific electrode. Here, we monitored the protons that are produced. A pH indicator method was developed using a hydrolytic defluorinase from *Delftia acidovorans* strain B in purified form or expressed recombinantly in *Pseudomonas putida* ATCC 12633. The method was also shown to be effective with *P. putida* F1 catalyzing oxygenative defluorination with α,α,α-trifluorotoluene. *P. putida* ATCC 12633 strains expressing different recombinant defluorinases showed differential growth and coloration on agar plates containing bromothymol blue and a fluorinated substrate. A purified defluorinase with a high pH optimum was assayed using the pH indicator *m*-cresol purple to identify six new substrates, one of which is a PFAS. LC-MS and fluoride electrode determinations require a single sample work-up and milliliter volumes. The proton monitoring methods described here can be performed in a microliter high-throughput format. It can also be used in solid matrices such as hydrogels. Although less rigorously quantitative than the single determination methods, rapid screening methods as described here are currently needed by researchers seeking to identify and characterize new microbes and enzymes able to biodegrade commercially relevant PFAS.

**IMPORTANCE:**

Fluorinated compounds are widespread as pesticides, pharmaceuticals, and legacy chemicals. Human health and ecosystem health problems arise from exposure to these chemicals. Currently, there is great interest in reducing exposure via bioremediation, and this is spurring efforts in screening for C-F bond-cleaving microbes and enzymes. C-F bond cleavage produces fluoride and a proton. Fluoride determination is difficult in many matrices and involves milliliter volumes and single-sample determinations. Here, proton release by enzymes and microbes was monitored on agar, in hydrogels, and in a microliter liquid high-throughput screening format. Six new substrates were identified for one microbial defluorinase enzyme.

## INTRODUCTION

Fluorinated compounds are widespread as pesticides, surfactants, heat-transfer agents, personal care products, and pharmaceuticals (1). Currently, the class known as per- and poly-fluorinated substances, or PFAS, is under close scrutiny for potential environmental and human health effects (2). Removing them from the environment, particularly drinking water supplies, is incurring great costs to companies, municipalities, and national governments (3). At present, remediation of fluorinated compounds uses physical and chemical processes, such as adsorption, incineration, or chemical defluorination processes (4).

Bioremediation could prove beneficial in terms of ease and cost, but currently suffers from a lack of suitable options to deal with many of the compounds of environmental concern (5). There is growing evidence for enzymes being able to cleave carbon-fluorine (C-F) bonds, even with -CF_2_ and CF_3_ moieties, but the range of substrates is currently limited (6, 7). This has led to a concerted effort in finding, characterizing, evolving, and engineering new C-F cleaving enzymes (8).

The success of finding or creating organisms and enzymes can be enhanced by the ability to rapidly screen the biodegradation of organofluorine compounds in a high-throughput manner. Methods such as liquid chromatography-mass spectrometry are rigorous but require extraction, workup, analysis by expensive equipment, and do single determinations (9). A more rapid and less demanding method is fluoride determination since C-F bond cleavage produces fluoride anion, due to the extreme electronegativity of fluorine (10). Fluoride has been determined by ion chromatography, ion-specific electrode, colorimetric assays, or biosensor-based methods (11, 12). The first two methods are not high-throughput, and the colorimetric methods suffer from a large number of interfering or adsorbing substances, limiting their usefulness (13, 14). It is also more difficult to determine fluoride in solid matrices, such as hydrogels and agar plates.

Many studies to measure microbiological or enzymatic defluorination rely on fluoride determination, as different mechanisms produce hydrogen fluoride (HF). Hydrogen fluoride is a surprisingly weak acid in water (pKa = 3.2) compared with other hydrogen halides that hold tightly onto an associated proton (15). Most biological studies are conducted at near-neutral pH, where HF will be in the dissociated form of a fluoride anion plus a proton. In that context, we considered here that proton detection might serve as a nearly universal method to follow defluorination in liquid media, on agar plates, and in hydrogels. Defluorination by hydrolytic, reductive, or eliminative mechanisms produces a proton in a 1:1 stoichiometry with fluoride anion (10, 15). This makes it possible to screen defluorination by these mechanisms using pH indicators.

The use of pH indicators in microbiological screening has a long history and has been used for physiological transformations that raise or lower pH (16). Different pH indicators have been used, depending upon the match between the pKa of the indicator and the starting pH of the medium (17). Many indicators are multicyclic phenols that have large extinction coefficients and characteristic colors (18). Sulfonephthalein pH indicators, such as bromothymol blue, phenol red, and *m*-cresol purple, have been used to determine proton production from enzyme reactions *in vivo* and *in vitro*. Notable examples are with nitrilases and esterases (19, 20). In those cases, carboxylic acids are produced that lower the pH, and the activity is determined using bromocresol green or methyl red. The activities of dechlorinating enzymes have been determined using several pH indicators, in both cuvettes and microtiter well formats (21, 22). Defluorinating enzyme activity has been reported using phenol red in a spectrophotometric assay (7). Bacteria expressing dechlorinating enzymes have also been screened on agar plates (23).

The present study significantly extends previous work by determining HF liberation in four media by chemical, enzyme, and whole-cell biodegradation formats and using the method to discover new defluorination reactions. Most notably, we report here activities with difluoro- and trihalo-carbon centers (Fig. S1). Differential defluorination activity was also demonstrated in agar plates and hydrogels, conditions under which fluoride release is difficult to determine. On agar plates, strains showing higher rates of fluoride production in liquid could be discerned on agar plates by faster rates of acidification as shown by rapid color change. Protocols were described to bind fluoride in hydrogels that stabilize and enhance cellular defluorination.

## RESULTS

### Optimizing indicators, conditions, and problem-solving for liquid screening near neutral pH

**Fig 1:**
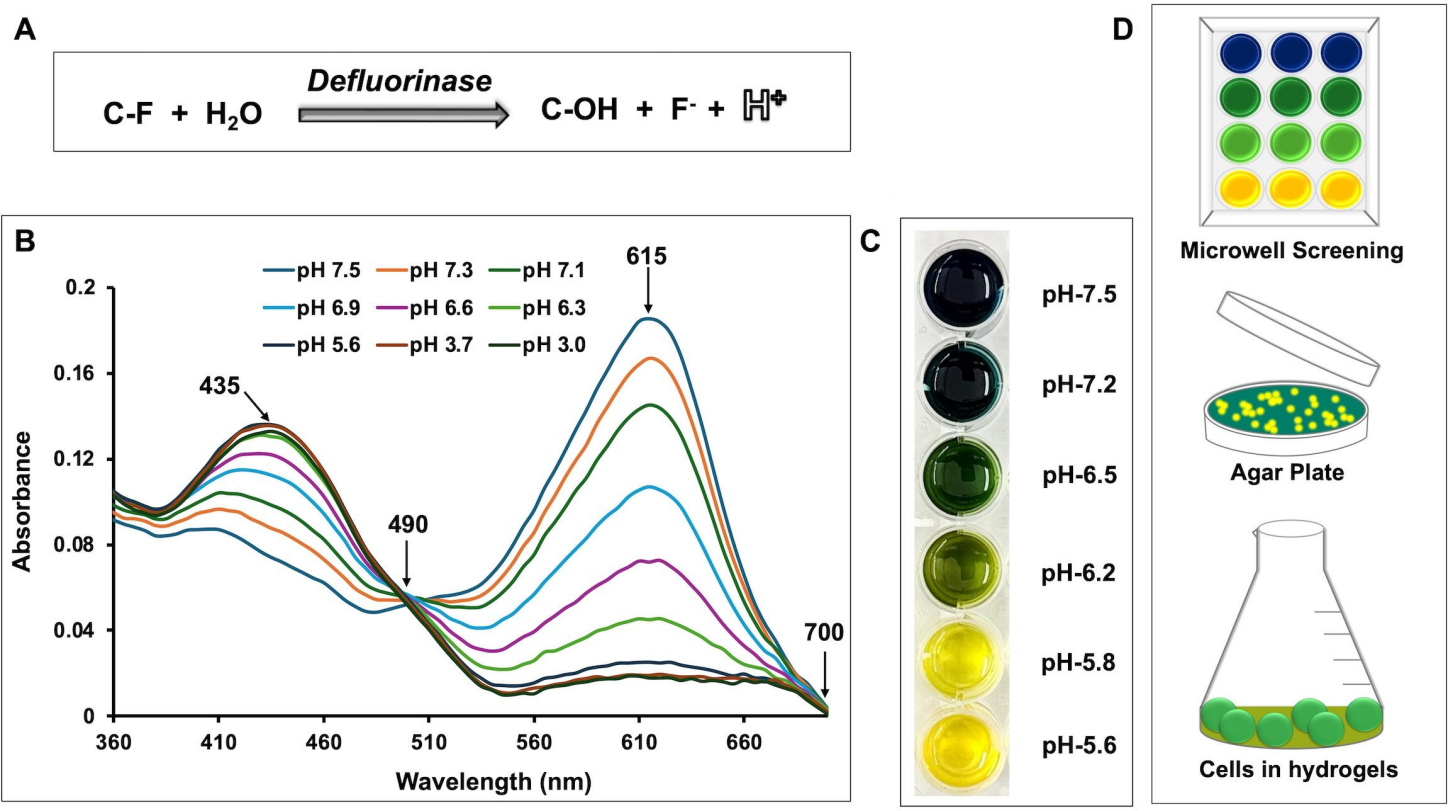
C-F bond cleavage by multiple mechanisms releases fluoride anion and a proton, the latter of which can be estimated spectrophotometrically, or visually in liquid, on agar, or in hydrogel beads. (**A**) Most defluorination reactions studied here were hydrolytic, and the general reaction stoichiometry is illustrated. (**B**) Shown is a spectral titration of bromothymol blue with protons from HCl showing a decrease in absorbance at 615 nm, an isosbestic point at 490 nm, and no contributing absorbance at 700 nm, allowing light scattering from cells to be determined simultaneously. (**C**) Visual observation of color changes as the pH decreases from 7.5 to 5.6. (**D**) Schematic showing the multiple utilities of the pH-screening method.

Most bacteria grow, and enzymes are optimally active, at near neutral pH, and hence, we focused our initial attention on pH indicators with pKa values ~ 7.0. Initial experiments used bromocresol purple (pKa ~6.4) and bromothymol blue (pKa ~7.0) and Tris and phosphate buffers. Both pH indicators were used at a starting neutral pH in microbial media and titrated with acid to determine relevant spectrophotometric values. Relevant determinations included: (i) the absorption maxima under acidic and slightly basic conditions, (ii) the isosbestic point during the transition, and (iii) a wavelength at which absorbance was baseline and thus usable to determine light scattering of cells to monitor growth concurrent with pH. After preliminary tests, bromothymol blue was selected for detailed studies. Fig. 1 shows titration curves for bromothymol blue, color change by eye, and the utility of the method for monitoring defluorination in different matrices. The acid maximum was at 435 nm, the isosbestic point was at 490 nm, the basic maxima was at 615 nm, and no absorbance was seen at 700 nm, permitting cell turbidity measurements.

We initially tested pH measurements of defluorination with 4-fluoro-1,3-dioxolan-2-one and lithium difluoro(oxalate) borate, a monofluoro or difluoro compound, respectively. Both compounds underwent spontaneous defluorination in neutral growth media and Tris-HCl buffer over a time course of hours, as determined by fluoride electrode (Fig. S2 to S4). The defluorination was similarly inferred by a decrease in pH using bromocresol purple or bromothymol blue.

Subsequently, an additional complexity to overcome was observed with microbes expressing defluorinase enzymes and tested with known substrates in microtiter well plates. The growth medium was initially poised at pH 7.5, where bromothymol blue (pKa ~7.0) is blue, and it was monitored over 1–2 days, as required for microbiological screening. In controls with only fluorinated substrates and without cells, a slight yellowing of the medium was observed, and the pH dropped to ~pH 6.5. The pH drop was close to the pKa of carbonic acid (6.4), and it was hypothesized to arise from the dissolution of carbon dioxide from the atmosphere. With actively growing cells, carbon dioxide from metabolism could also contribute to pH lowering. To mitigate this problematic acidification, it was found that adding 200 μL of 5 M NaOH to the empty wells of microtiter well plates decreased the background pH change (Fig. S5). The NaOH trapped much of the carbon dioxide but did not impact protons in the middle wells derived from microbe-catalyzed defluorination.

### Microbiological defluorination in liquid media

**Fig 2:**
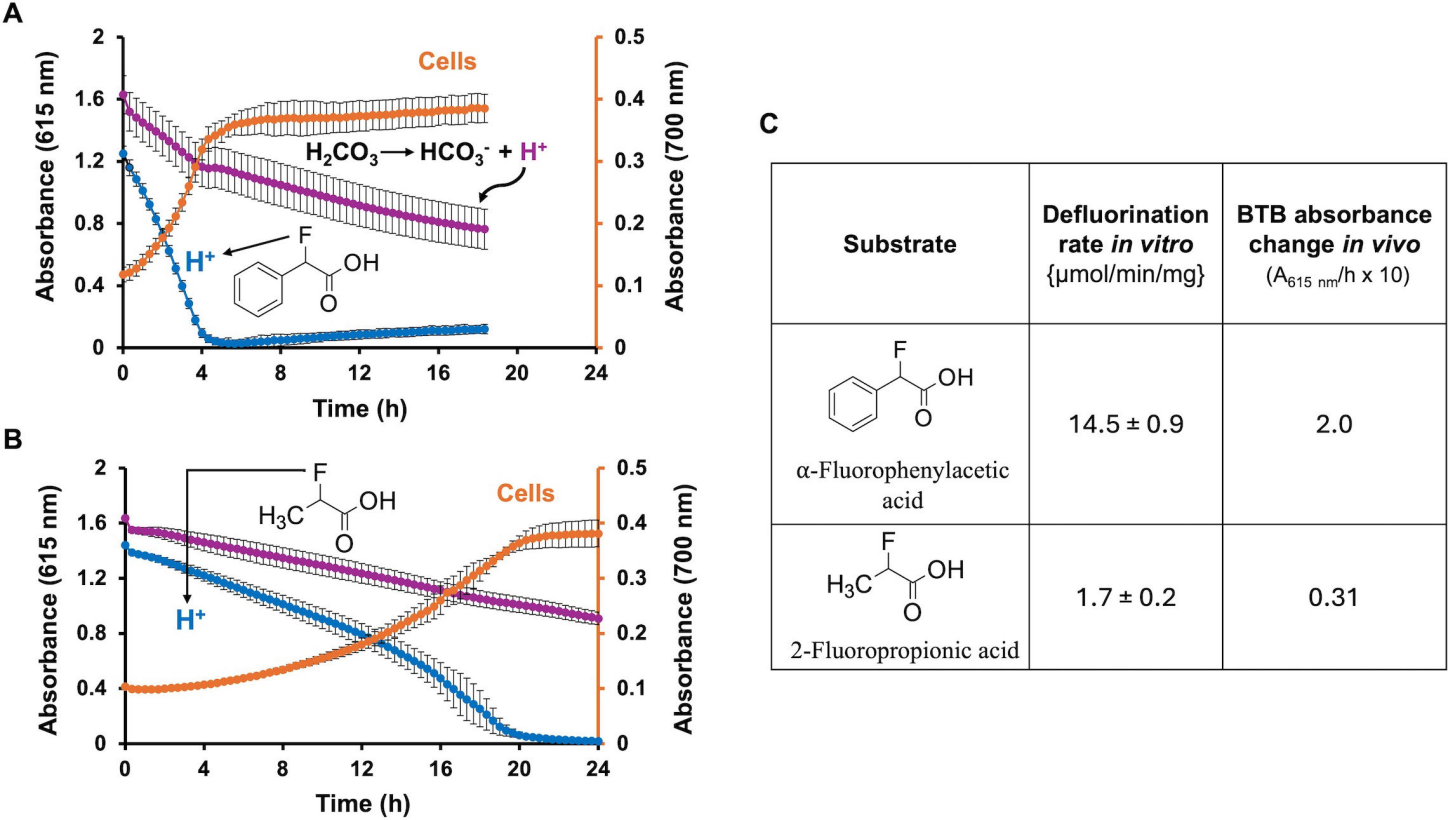
Measuring substrate defluorination *in vivo* by pH change using a recombinant *Pseudomonas* expressing a defluorinase, DEF1. The reactions and cell growth were monitored by changes in bromothymol blue absorbance at 615 nm and turbidity at 700 nm (OD_700_), respectively. The blue curves show the change in pH as a drop in absorbance at 615 nm when cells are present. The magenta curve shows absorbance at 615 nm with substrate alone, which controlled for the dissolution of carbon dioxide from the atmosphere, which gives background acidification. The orange curve shows the increase in OD_700_ due to cell growth. (**A**) Reactions and growth with α-fluorophenylacetic acid. (**B**) Reactions and growth with 2-fluoropropionic acid. (**C**) Comparison of the rates of defluorination measured in a previous study with purified enzyme *in vitro* with the *in vivo* rate determined here via acidification by the change in absorbance at 615 nm. The rate was determined from the linear regions of the blue curves. The background absorbance change (magenta curve) was subtracted to correct for the control pH change. The rates were multiplied by 10 to make the numeric comparison easier. Error bars shown in parts A and B represent standard deviations from triplicate determinations.

Two substrates were used for which the *in vitro* rates of the defluorinase were known to vary by ~8-fold (24) (Fig. 2). Using the same strain expressing the same level of defluorinase allowed testing of the discriminating power of the pH assay. As shown in Fig. 2, it was possible to see a pH change for both and a variation in the response.

α-Fluorophenylacetic acid and 2-fluoropropionic acid had previously been shown to be substrates for the defluorinase from *Delftia* sp. (DEF1), which was expressed here constitutively in *P. putida* ATCC 12633. *P. putida* 12633 has been shown to grow on both as a sole carbon source. The absorbance at 615 nm declined at a nearly linear rate over a 4 h time course with α-fluorophenylacetic acid (Fig. 2A). There was significant, but correctable, acidification in the substrate control (with indicator), ostensibly due to carbon dioxide derived from cells and the atmosphere. The backgrounds ranged from 20% to 40% of the total rate of pH decrease, and that was used to correct the substrate-dependent rate. Turbidity measurement at 700 nm indicated one cell doubling over the 4 h time course.

With 2-fluoropropionic acid (Fig. 2B), the substrate-dependent rate was lower, as expected based on prior determinations of *in vitro* defluorination rates using purified enzyme. Acidification was observed as the decrease of the 615 nm peak over 20 h, and the cells underwent approximately two doublings during that time. The relative rates of proton release in whole cells could be compared to fluoride release rates (Fig. 2C). For the latter, α-fluorophenylacetic acid is defluorinated 8.5-fold faster than 2-fluoropropionic acid by DEF1. Here, the rate of acidification was 6.5-fold faster with a-fluorophenylacetic acid than with 2-fluoropropionic acid. These data indicate that the more convenient and continuous pH assay is useful for relative comparisons of substrate defluorination.

### Screening oxygenative defluorination of a trifluoromethyl group by wild-type *P. putida* F1

The previous experiments used pH changes to follow proton release in reactions catalyzed by a heterologously expressed hydrolytic defluorinase. Here, we expanded the utility of the assay to a native microorganism previously shown to catalyze defluorination by a completely different mechanism and with a substrate containing a trifluoromethyl group (13). *P. putida* F1 uses an oxygenase to incorporate dioxygen into aromatic rings, leading to unstable intermediates that undergo defluorination (24, 25).

**Fig 3:**
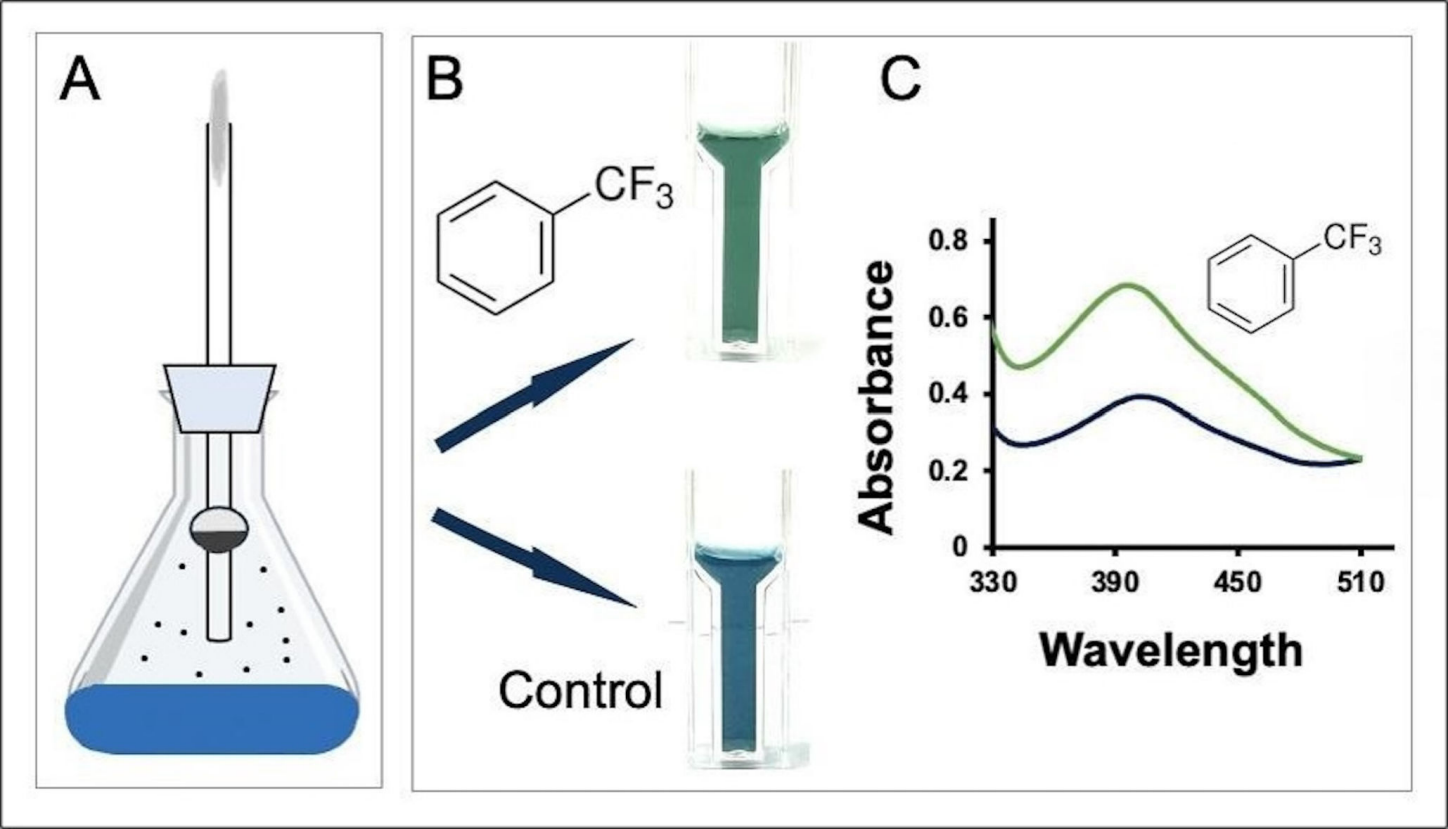
Using bromothymol blue to show defluorination and coincident acidification by wild-type *P. putida* F1 with induced toluene oxidizing enzymes. (**A**) Schematic of a shake flask culture in which a volatile carbon and energy source is provided in the vapor phase via a bulb with a single opening suspended above the medium. (**B**) Photographs showing visible color differences in supernatants from cultures that were grown on toluene and then incubated overnight with a vapor bulb containing α,α,α-trifluorotoluene (top, structure shown) or an empty bulb (bottom, control). (**C**) Partial absorbance spectra of the supernatants shown in panel B, showing a greater increase of the acidic form of bromothymol blue with cells incubated with α,α,α-trifluorotoluene (green) vs the empty vapor bulb (blue).

The experiment used volatile α,α,α-trifluoro-toluene that can be supplied via a vapor bulb suspended above the liquid medium after growing the cells on toluene to induce the toluene-oxidizing enzymes (Fig. 3A). A parallel toluene-grown culture that was not exposed to α,α,α-trifluorotoluene was included as a control. In the presence of the fluorinated substrate, 1.1 mM fluoride was detected, but no fluoride was found in the control. The mechanism of defluorination was previously established, and it is known to produce protons and fluoride in a 1:1 ratio (13). Acidification of the medium could be ascertained both visually (Fig. 3B) and by spectrophotometry via the increasing absorbance at 400 nm (Fig. 3C). The level of defluorination and proton formation via this native strain was an order of magnitude less than what was seen with the recombinant *P. putida* 12633 strain expressing DEF1 and reacting with α-fluorophenylacetic acid, which turned the pH indicator a bright yellow. These observations indicate that levels of defluorination of ~10 mM substrate are easily observed, and ~1 mM can be detected with overnight incubations. Levels of defluorination in the micromolar range are unlikely to be discernible via the methods described here. The control reaction (cells incubated with an empty vapor bulb) gave no change in the absorbance spectrum of the pH indicator over the 1-day time course of the experiment.

### Defluorination on agar plates

The pH indicator was incorporated into agar plates to investigate defluorination by recombinant *P. putida* ATCC12633 strains by easy visual inspection (Fig. S6). Previous efforts had subjected strains expressing either DEF1 or a different defluorinase from a *Dechloromonas* species (DEF2) to adaptive laboratory evolution (ALE) (26). Initial unadapted recombinant strains grew poorly in liquid cultures due to fluoride stress (27), but ALE using α-fluorophenylacetic acid as the sole carbon and energy source led to more rapid growth and defluorination.

**Fig 4:**
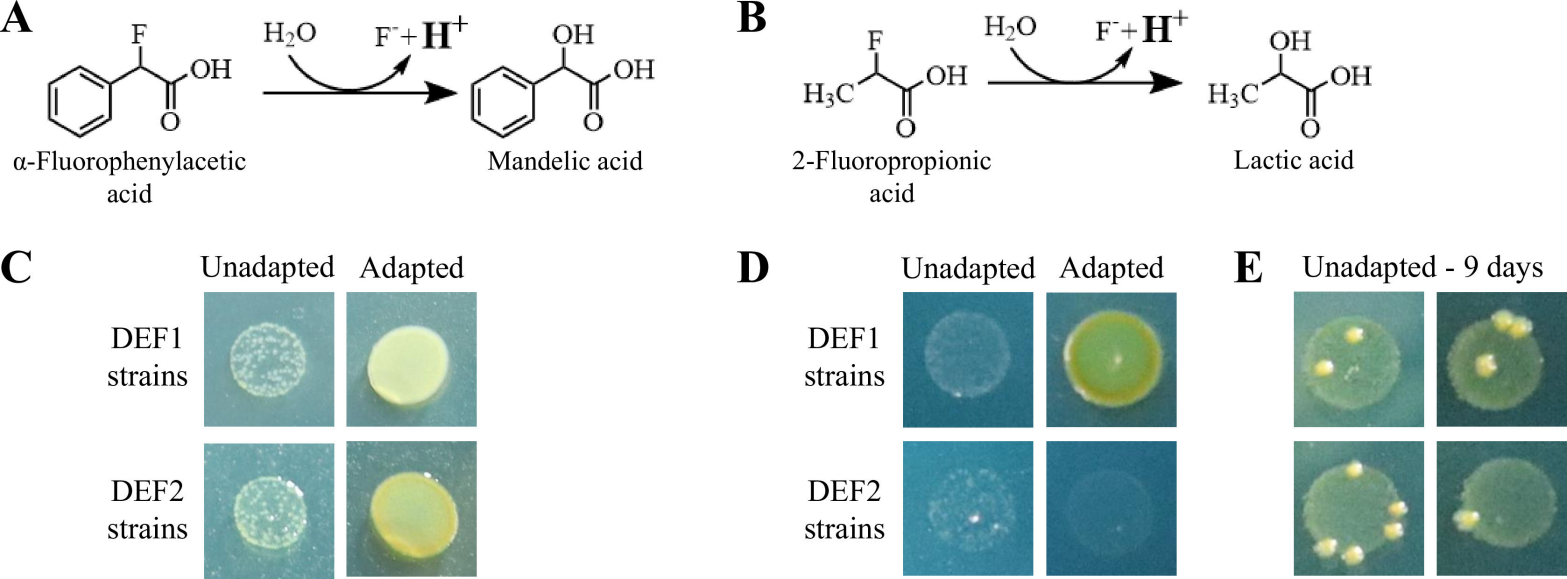
Determining C-F bond cleavage by acidification in spotted cell patches on agar plates containing bromothymol blue. Four different recombinant *P. putida* ATCC 12633 strains were used and contained either defluorinase (DEF) one or two, and each was either in a strain that had been adapted to α-fluorophenylacetic acid or not, as previously described (26). The spotted suspensions contained ~4 × 10^5^ cells in all cases. (**A**) Defluorination reaction scheme for α-fluorophenylacetic acid, (**B**) Defluorination reaction scheme for 2-fluoropropionic acid. Photographs were taken of the four strains growing on plates containing (**C**) α-fluorophenylacetic acid (2-day incubation) or (**D**) 2-fluoropropionic acid (3-day incubation). (**E**) Instead of growing confluently, small, discrete colonies of the unadapted DEF2 strain grew within the original spot after 9 days on 2-fluoropropionic acid plates.

Here, liquid suspensions of adapted and unadapted strains expressing DEF1 or DEF2 were spotted in small volumes onto bromothymol blue agar plates with either α-fluorophenylacetic acid (Fig. 4A and C) or 2-fluoropropionic acid (Fig. 4B through D). With α-fluorophenylacetic acid, only a small fraction of unadapted cells grew in 2 days, and the yellow color was faint, consistent with previous studies in liquid cultures that had shown inhibited growth and lower levels of defluorination (27). The adapted cells that were spotted showed confluent growth and a strong yellow color. In Fig. 4D, similar results were seen with DEF1, but no significant growth was observed with the DEF2 unadapted or adapted cells after 3 days. This might be due to the slower defluorination rate with 2-fluoropropionic acid by the DEF2 strain (26).

Although growth after 3 days was negligible for the unadapted DEF2 strain, we observed a significant outgrowth of 1–5 colonies in the spot after 9 days, and they were moderately yellow (Fig. 4E). The characteristics of the eleven colonies observed are beyond the scope of this methods paper, but this observation shows the potential to use pH indicator plates for adaptive evolution experiments. In total, the plating experiments show the value of measuring pH changes on agar plates to investigate growth rate, defluorination, and potential adaptation using different fluorinated growth substrates.

### Determining defluorination in hydrogels

**Fig 5:**
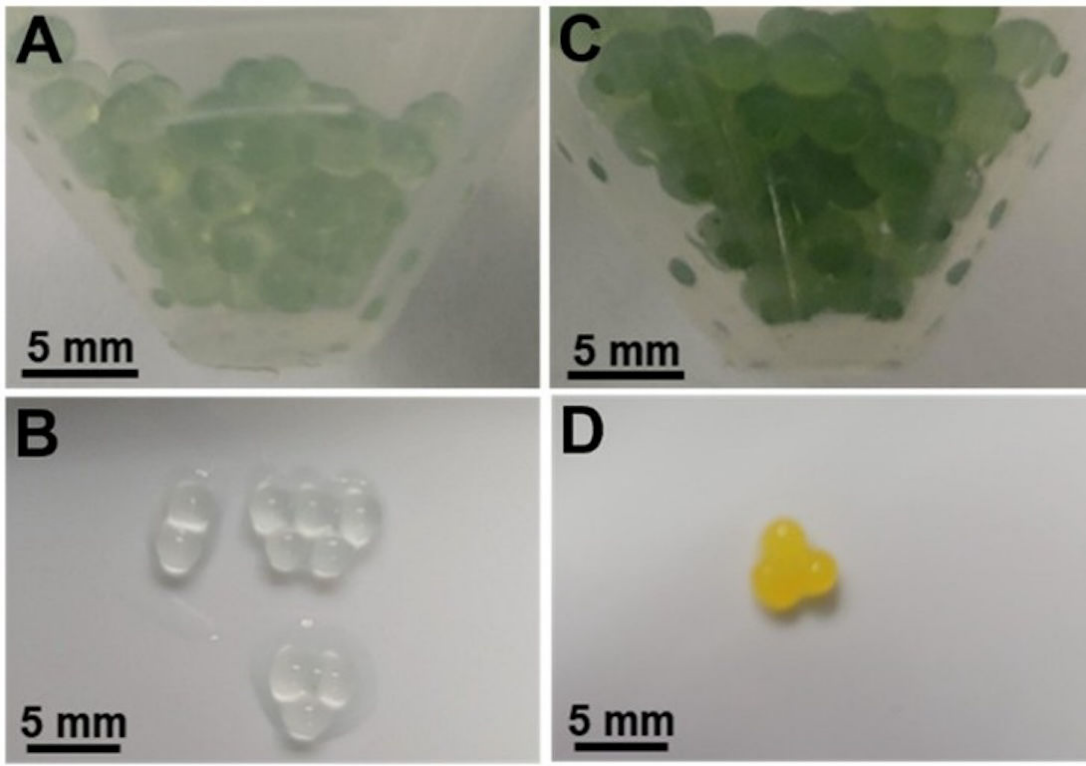
Alginate hydrogels cross-linked with calcium were suspended in liquid containing bromothymol blue (left) or containing interned cells (right). Photograph at the bottom of the beads exposed to 40 mM α-fluorophenylactic acid for 24 h.

Immobilized cells have been used to remove pollutants from water, and alginate has been a widely used matrix for cell entrapment (28). The goal here was to determine defluorination via proton release by cells in a solid matrix while simultaneously binding fluoride anion to co-encapsulated metals. In initial experiments, Ca-alginate beads were made with bromothymol blue incorporated into the beads during polymerization. Incubation of the beads with α-fluorophenylacetic acid in weakly buffered bromothymol blue solution led to a change from green to a distinct yellow color in the beads containing cells. Alginate is hydrophilic, but cell membranes would be expected to retain the hydrophobic dye bromothymol blue. Beads without cells were colorless. The dye readily diffused out of the gels lacking cells, indicating that the hydrophobic dye was at least partially retained by the presence of the cells. This is consistent with the previously observed concentration of the dye in the colonies on agar plates (Fig. 5).

In follow-up experiments, the weakly buffered α-fluorophenylacetic acid solution surrounding cells encapsulated in calcium alginate was tested for free fluoride. The detected fluoride was only 18% of the level found in a parallel incubation using free cells instead of encapsulated cells, but the final measured pH was similar. This suggested that the Ca-alginate was sequestering fluoride, but not the concomitantly released protons. It is well known that fluoride binds avidly to calcium and that CaF_2_ is highly insoluble. Despite this, there was no significant weakening of the gel structure observed. Since fluoride is known to be highly toxic to biodegrading cells, the binding of fluoride can be beneficial for cells by lowering fluoride equilibria and preventing binding to intracellular metal centers. Moreover, the pH decrease that could be observed visually from the yellow color shows that while free fluoride is difficult to determine in such a protective milieu, the co-released protons can serve as a better monitor for defluorination.

**Fig 6:**
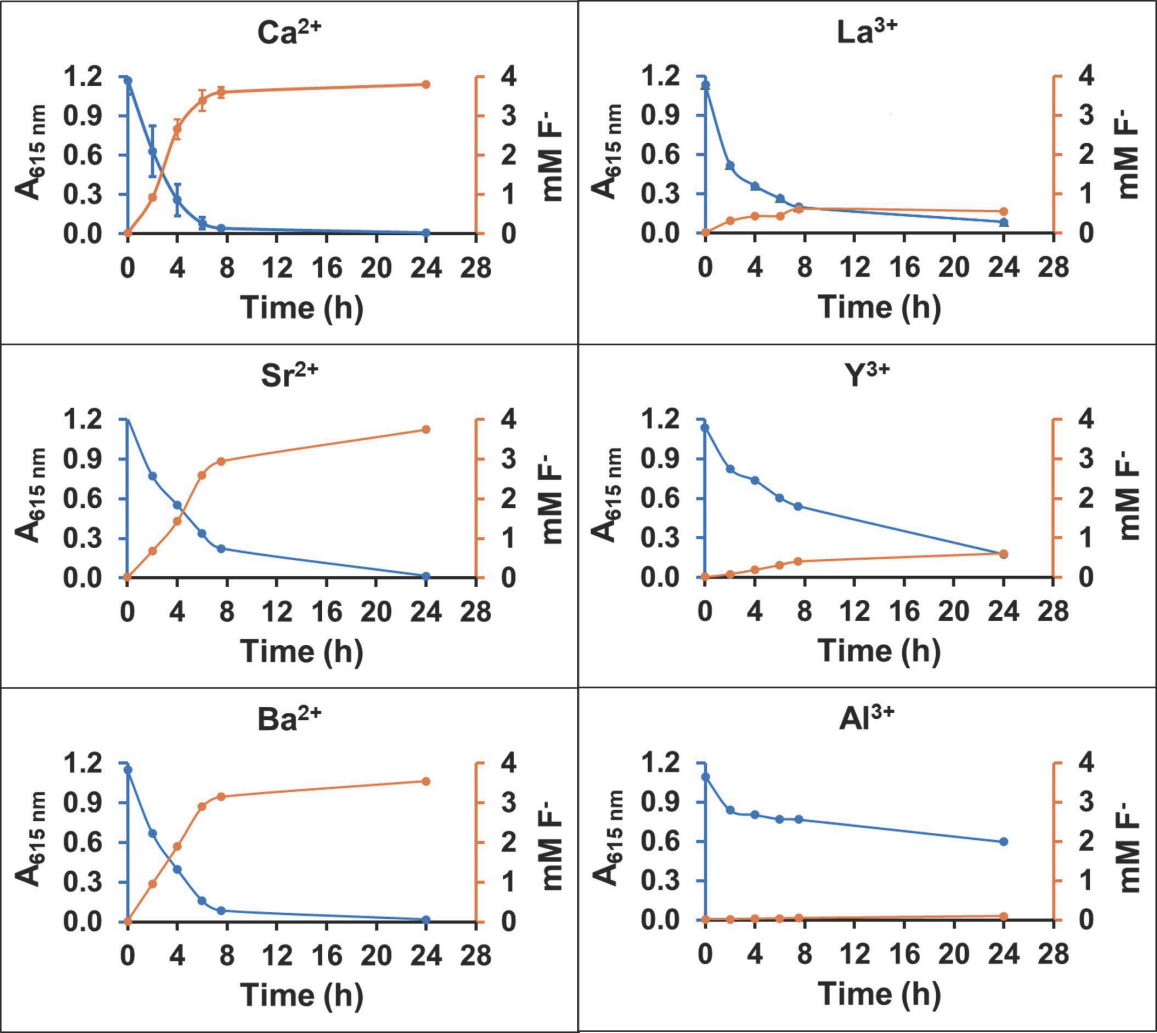
Measuring defluorination *in vivo* by a recombinant *Pseudomonas* expressing a defluorinase encapsulated in alginate cross-linked by different metals that can potentially coordinate fluoride anion. Defluorination was monitored via the accumulation of protons in the liquid surrounding the beads by the drop in absorbance of bromothymol blue at 615 nm (blue). Fluoride anion was measured in the liquid using an ion-specific fluoride electrode (gold). Alginate gel data are also shown in Fig. S7.

Subsequently, alginate gels were made with different metal cations, some of which had previously been shown to sequester fluoride (29, 30). In that context, we next conducted experiments with cells encapsulated in alginate beads prepared using barium, calcium, strontium, aluminum, lanthanum, or yttrium as the crosslinking metal ions (Fig. 6). The relative defluorination of 2-fluoropropionic acid by recombinant *P. putida* 12633 in the beads was compared by analyzing the surrounding buffer for a decrease in pH as determined by a decrease in absorbance at 615 nm. The release of fluoride into the buffer was also determined using a fluoride electrode as described in the methods.

Cells encapsulated in alginate beads cross-linked with calcium, strontium, barium, or lanthanum all showed a significant release of protons in 8 h, indicating a significant amount of defluorination (Fig. 6). Cells in yttrium alginate showed a slower decrease in absorbance at 615 nm, and aluminum alginate showed the lowest rate of acidification. Aluminum and yttrium are known to be toxic to *Pseudomonas* spp., and that may underlie the lower degree of acidification. Calcium, strontium, barium, and lanthanum also showed a nearly reciprocal appearance of fluoride anion in the liquid medium. Lanthanum supported significant defluorination and the lowest amount of fluoride released into the surrounding medium. Lanthanum is known to bind fluoride very avidly (31). Yttrium is also known to complex with fluoride, although not as tightly as lanthanum (32). Controls of the assay solutions incubated without beads showed no significant pH change during incubation, and no fluoride was detected, indicating that the substrate did not undergo spontaneous defluorination. We did not analyze fluoride bound to metals, which requires extraction as gaseous HF, and our laboratory is not set up to capture and analyze this corrosive gas (33). However, based on the observed disappearance of substrate and levels of fluoride release, the lanthanum-alginate gel showed the best biodegradation coupled with fluoride sequestration, consistent with the known avidity of lanthanum for fluoride anion (30).

### Screening purified enzymes to identify new substrates

Experiments described above used known substrates of a defluorinase (DEF1) and an oxygenase to show the utility of pH indicators to determine defluorination (Fig. 2 to 6). Next, we used the pH method to screen for potential new substrates of DEF1 (Table 1). Reactions were initially monitored using conditions developed for cell-based defluorination (Fig. 2 and 4). However, using bromothymol blue with a starting pH of 7.5, which was ideal for cells, was found to be sub-optimal when used with purified DEF1 enzyme that has the highest activity at its pH optimum of 8.5–9.5.

**TABLE 1:** Testing activity of purified DEF1 on fluorinated compounds^*a*^

Chemical tested	Relative rate by pH^*b*^	F^-^ electrode assay^*c*^ (µmol/min/mg)
Previously known substrates		
⍺-Fluorophenylacetic acid	+++	14.5
2-Fluoropropionic acid	+++	1.7
Chlorofluoroacetic acid	++	0.13
New substrates		
⍺-Chlorophenylacetic acid	+++	No fluoride detected
Chloroacetic acid	+++	No fluoride detected
2-Fluoro-2-phenoxyacetic acid	++	0.83
2-Fluoro-2-(trifluoromethoxy) acetic acid	++	0.16
Bromofluoroacetic acid	+	0.09
Bromodifluoroacetic acid	+	0.01
Not a substrate		
1-Fluoro-cyclopropane-carboxylic acid	-	-

^*a*^ In 2 mM Tris (pH 8.5) with 0.2 mM *m*-cresol purple. Relative activities determined by pH shift are compared with activity determined from fluoride release for fluorinated substrates.

^*b*^ Defined in Materials and Methods.

^*c*^ Fluoride release was determined by fluoride-specific electrode as described in the methods. Units are per mg enzyme. Could not be determined for chlorinated substrate.

To adjust to the pH optimum of the enzyme, we selected *m*-cresol purple, a different sulfonephthalein pH indicator (34). We first titrated *m*-cresol purple with acid under the conditions at which we would assay the enzyme (2 mM Tris buffer, starting pH = 9.5) (Fig. S8), since several different pKa values have been reported in the literature (35, 36) and it is important to match the pKa of the buffer to the pKa of the pH indicator (37). In that context, we determined the extinction coefficient and made a standard curve for proton concentration versus absorbance. Although pH changes can be determined theoretically from the Henderson-Hasselbalch equation, previous enzyme studies indicated that determining proton formation experimentally from a standard titration under precise conditions is more accurate (38). After standardization, substrate transformation rates could be estimated by proton formation and compared with data on fluoride formation.

We found that the pH screening method was effective in identifying new substrates rapidly and estimating relative rates (Table 1; Fig. S9). In separate studies, to be discussed elsewhere, the DEF1 enzyme showed order of magnitude different rates depending on buffer, buffer concentrations, and pH. This complex behavior may relate to the known half-site reactivity of fluoroacetate dehalogenase (39) and will be studied in further detail in subsequent work.

Despite these caveats, the rapid pH screening method described here identified new substrates (Table 1). Six new substrates for the fluoroacetate dehalogenase are reported here using the pH assay (Table 1). For those containing fluorine substituents, activities were confirmed using a fluoride-specific electrode. Previously, we had shown reactivity with α-fluorophenylacetic acid, 2-fluoropropionic acid, and chlorofluoroacetic acid (27).

## DISCUSSION

Microbiological defluorination is of particular interest with respect to numerous ongoing efforts to biodegrade fluorinated pesticides, surfactants, and legacy chemicals, many of which are designated as PFAS. Such efforts will require the use of diverse screening methods, often high throughput, that might be in homogenous liquids, droplets, or other materials (8). The pH indicator methods described here offer a way to rapidly screen in microliters of liquid, hydrogel beads, agar plates, or other materials. The pH measurement can then be corroborated by more time-consuming and sample-intensive methods such as NMR, liquid chromatography, and high-resolution mass spectrometry (40–42).

pH indicators have been used in numerous microbiological media and enzyme assays, but have not been used extensively to study microbial defluorination. The present paper was designed to fill that gap. Defluorination via hydrolytic, reductive, or eliminative mechanisms is known to release both fluoride anion and a proton (10, 15). Most studies use fluoride determination as a measure of defluorination, but complex media ingredients may sequester fluoride, and solid phase systems generally prohibit fluoride measurement in the matrix. Potential methods to overcome matrix effects would be to determine fluoride via an engineered fluoride riboswitch or fluoride aptamer *in vivo* (11, 43).

In the present study, we sought to develop a broadly applicable method of determining defluorination *in vivo* or *in vitro* by determining the release of protons that are stoichiometrically released with fluoride anions. Proton release causes acidification that can be determined in liquids, agar plates, and with encapsulated cells. The latter may be particularly useful for the deployment of bacteria degrading C-F compounds, where it can be desirable to sequester fluoride to protect against toxicity (44).

Multiple defluorination processes were tested here to determine the broad utility of pH indicator methods. They include: (i) abiotic defluorination reactions in buffer, (ii) recombinant cells in liquid culture, which express a hydrolytic defluorinase enzyme, (iii) a native bacterium in growth medium expressing oxygenase and dehydrogenase enzymes that mediate defluorination of a -CF_3_ group, (iv) bacteria with different properties spotted onto agar plates, (v) bacteria entrapped in a hydrogel matrix, and (vi) a purified enzyme that was screened to identify new substrates. The formats in which the systems were tested were shake flasks, microtiter well plates, agar plates, and hydrogel beads. Previously, most studies using pH indicators for dehalogenation have been conducted to study dechlorination reactions *in vivo* and *in vitro* by monitoring the formation of HCl (21, 22, 45). Most experiments described here were based on HF production, but mixed substrates with chlorine or bromine with fluorine were also used.

It is important to point out caveats for using the methods reported here. First, the drop in pH requires the use of a low buffering capacity of the medium. This can change the pH environment sufficiently to cause lower reaction rates with cell or cell-free systems. Hydrogen fluoride, with a pKa of 3.2, is only a moderately strong acid in aqueous environments (10). However, a pH drop from near neutral to pH five resultng from HF liberation mediated by the activiity of microbial cells or enzymes is measurable with available pH indicators that are suitable for use within that pH range.

Second, it is possible that some bacteria might transform the pH indicator, or it may be toxic. We did not observe evidence for either with the *Pseudomonas* sp. used in this study, but other microorganisms may be more sensitive to chemical additives.

Third, although pH indicators can theoretically quantify proton release, strict quantification of enzyme activity in this manner is difficult in practice. Although the Henderson-Hasselbalch equation can theoretically be used to quantify protons, biological mixtures present many substances that are hard to account for completely. Especially in whole cell assays, many factors are changing simultaneously. These include the consumption of an acidic substrate, the dissolution of carbon dioxide to produce bicarbonate, and the potential release of other buffering organic materials from cells. The methods described here in solid media are best used in a semi-quantitative manner. Examples of this utility are in comparing different substrates or perhaps in screening experiments to test variant enzymes. On agar plates, the method is useful to compare strains. This was shown by distinguishing color formation on agar plates using strains previously characterized and known to have differential defluorinating activities. Hydrogel beads could be used with dye in the gels or in the surrounding media.

Finally, the pH indicator assays described here are most suitable for studies using moderate-to-high substrate concentrations and for relatively robust rates of defluorination. Other studies determining PFAS biodegradation in environmental samples at ambient concentrations of parts per billion (ppb) or lower may require higher sensitivity methods such as liquid chromatography-mass spectrometry. Similarly, laboratory studies using part per million (ppm) level substrate concentrations may require the use of a fluoride-specific electrode. The methods described here are most useful as a rapid, low-volume screen in diverse media that can be applied in a high-throughput manner.

## MATERIALS AND METHODS

### Chemicals

Chemicals were purchased from MilliporeSigma (Saint Louis, MO, USA) or as indicated: (*RS*)-α-fluorophenylacetic acid, 2-fluoro-2-(trifluoromethoxy)acetic acid, and 2-fluoro-2-phenoxyacetic acid (Enamine, Kyiv, Ukraine); (*RS*)−2-fluoropropionic acid, (*RS*)-α-chlorophenylacetic acid, 2,2-difluoro-2-(pyridin-2-yl)acetic acid, bromofluoroacetic acid, 1-fluorocyclopropanecarboxylic acid, potassium 2-bromo-2,2-difluoroacetate, and lithium difluoro(oxalato)borate (Ambeed, Arlington Heights, IL, USA); bromothymol blue sodium salt, (*R*)-hydroxypropionic acid (D-lactic acid), α,α-difluorophenylacetic acid, and difluoroacetic acid (Oakwood Chemical, Estill, SC, USA); chlorofluoroacetic acid (abcr GmbH, Karlsruhe, Germany); 2-chloro-2,3,3,3,3-tetrafluoropropanoic acid (A2B Chem, San Diego, CA, USA); α,α,α-trifluorotoluene, yttrium chloride, and tris base (ThermoFisher, Rockford, IL, USA); HEPES (GoldBio, St. Louis, MO, USA) and bromocresol purple (Matheson, Coleman & Bell, Norwood, OH, USA). The structures of the chemicals tested in assays, along with their associated names, are shown in Fig. S1.

### Strains, media, growth conditions, and enzymes

*P. putida* ATCC 12633 strains constitutively expressing recombinant defluorinases from *Delftia acidovorans* Strain B (DEF1) (46) or *Dechloromonas aromatica* RCB (DEF2) (7), which were either unadapted or adapted to growth on α-fluorophenylacetic acid, were described previously (26, 27). A variant of the unadapted DEF1 expression strain that also constitutively expressed the fluoride/proton antiporter from *P. syringae* (CLC^F^-psy) (47) from plasmid pSW002-*P*_psba_ (48) was also used and will be described in detail elsewhere. *P. putida* F1 was acquired previously from David Gibson (49).

Cultures in liquid media were grown at 28°C or 30°C in baffled shake flasks or in 96-well flat bottom suspension culture microplates (Genesee Scientific, El Cajon, CA, USA), respectively. *P. putida* 12633 cells for pH shift assays were grown in Miller lysogeny broth (LB) (BD Biosciences, Franklin Lakes, NJ, USA) or normal (40 mM phosphate buffer) Mineral Salts Broth (MSB) (50) defined medium with 50 µg/mL kanamycin sulfate (DEF1 or DEF2) or with 20 µg/mL tetracycline hydrochloride also added (DEF1 + CLC^F^ psy). Either 25 mM mandelic acid or 50 mM D-lactic acid was added to the normal MSB as the sole carbon and energy source as appropriate. Growth assays in microwell plates were done in weakly buffered MSB (5 mM phosphate buffer) without antibiotics and containing either α-fluorophenylacetic acid or 2-fluoropropionic acid as carbon and energy sources at the indicated concentrations and 0.1 mM bromothymol blue sodium salt. *P. putida* F1 cells used in pH shift assays were grown without antibiotics in normal MSB, with toluene delivered continuously in the vapor phase as the sole carbon and energy source. Solid MSB assay media had 1.5% noble agar (BD Scientific) added.

The purification of six-histidine tagged DEF1 from the *P. putida* 12633 host via immobilized-metal affinity chromatography (IMAC) using a GE Healthcare (Cytiva, Marlborough, MA, USA) AKTA fast liquid protein chromatography (FPLC) system and a HisTrap HP 5 mL column (Cytiva) charged with Ni^2+^ was described previously (27).

### Fluoride measurements with an ion-specific electrode

Free fluoride was measured with a Fluoride Ionplus Sure-Flow Combination ISE fluoride electrode that was interfaced with an Orion Start A214 pH/ISE meter (Thermo Fisher) and calibrated with sodium fluoride (NaF) standards of appropriate ranges prepared in DI water. Per the manufacturer’s instructions, 500 µL of each sample or calibration standard was combined with 500 µL of total ionic strength adjustment buffer (TISAB) (58.5 g/L NaCl, 15 g/liter acetic acid, 66 g/L sodium acetate, and 1 g/L 1,2-cyclo-hexane diaminetetraacetic acid) prior to insertion of the electrode for fluoride determinations.

### Standardization of bromothymol blue, bromocresol purple, and m-cresol purple by acid titration for spontaneously defluorinating chemicals and biological assays

Bromothymol blue (0.007 mM) or bromocresol purple (0.015 mM) was added to the mineral salt basal (MSB) medium, with the pH adjusted to 7.5. The *m*-cresol purple solution was prepared at 0.026 mM in 2 mM Tris-HCl buffer (pH 10). The stock concentrations of bromothymol blue, bromocresol purple, and *m*-cresol purple were 0.15 mM, 0.74 mM, and 1.3 mM, respectively, with each prepared in deionized water. Titrations were done by adding measured amounts of 0.5 N HCl dropwise to the indicator solutions, and pH was measured with an Oaktron Instruments pH electrode (Environmental Express, Charleston, SC) interfaced with an Accumet Basic pH meter (ThermoFisher). Absorbance readings and spectra were acquired using an Agilent Cary UV-visible spectrophotometer (Santa Clara, USA) at the indicated wavelengths in polystyrene semi-micro cuvettes (1 cm pathlength) (Sarstedt, Nümbrecht, Germany).

Correlation of abiotic fluoride release with pH decrease was assayed by adding different concentrations (5–50 mM) of 4-fluoro-1,3-dioxolan-2-one or lithium difluoro(oxalato) borate into 10 mM Tris-HCL (pH 7.5) or normal MSB (pH 6.8) containing 6 μM bromothymol blue or 10 μM bromocresol purple, stirring at room temperature using a magnetic stirrer, and measuring pH with a pH electrode and meter as above . Free fluoride was measured as described above, and color changes were observed visually.

A standard curve correlating pH change with color change was done by adding measured amounts of 0.01 N HCl to aliquots of 0.2 mM *m*-cresol purple in 2 mM Tris-HCl (pH 9.5) prepared from a 1.3 mM stock solution in DI water. Absorbance at 585 nm was measured in a Tecan Infinite M Nano Plus microplate reader/shaking incubator (Männedorf, Switzerland) and then plotted vs [HCl].

### Whole cell pH shift defluorination assays

Assays with *P. putida* ATCC 12633 recombinantly expressing DEF1 and CLC^F^-psy were conducted in 96-well plates using the Tecan microplate reader/shaking incubator. Assays were done in 0.200 mL aliquots of weakly buffered MSB (pH 7.5) containing 0.1 mM bromothymol blue and ⍺-fluorophenylacetic acid or 2-fluoropropionic acid at the indicated concentrations. Aliquots of concentrated cell suspensions were added to a working cell density that was equivalent to an absorbance at 600 nm (OD600 nm) of 0.1 as measured in a 1 cm pathlength cuvette using a Cary 60 spectrophotometer. The assay plates were incubated at 30°C with linear (197 s) followed by orbital (991 s) shaking at 1 mm amplitude. Changes in bromothymol blue absorbance or cell density were tracked at 615 or 700 nm, respectively.

To assay for defluorination of α,α,α-trifluorotoluene by wild-type *P. putida* F1, single colonies of the strain were transferred from an LB agar plate into separate 20 mL aliquots of normal MSB in 250 mL baffled flasks and incubated overnight on a shaker at 200 rpm with toluene provided as the sole carbon and energy source via a vapor bulb suspended inside each flask. Aliquots of the overnight cultures were transferred into a fresh 20 mL aliquot of the same medium to an OD600 nm = 0.1, incubated with the toluene vapor bulbs until the cultures were at OD600 nm = 0.5, and then harvested by centrifugation at 4,100 × *g*. The cell pellets were each washed 1× with 1 vol of phosphate-free MSB (pH = 6.8) and resuspended in 20 mL aliquots of weakly buffered MSB (pH = 7.5). The cell suspensions were transferred into sterile 250 mL flasks, and bromothymol blue was added to 0.1 mM. A new vapor bulb containing α,α,α−trifluorotoluene was added to one flask (experimental), an empty vapor bulb was added to the other flask (negative control), and the assays were incubated overnight with shaking as above. Aliquots of cells from each flask were transferred into sterile 1.7 mL polypropylene microcentrifuge tubes (Dot Scientific, Burton, MI), centrifuged at 8,600 × *g* for 5 min, and then aliquots of the supernatants were assayed for free fluoride as above or scanned from 300 to 800 nm with a Cary 60 spectrophotometer to show the relative amounts of the basic or acidic forms of bromothymol blue at 615 or 435 nm, respectively, in the experimental treatment vs the control.

### pH shift assays on agar plates

Weakly buffered pH indicator plates were prepared with 13 μM bromothymol blue and α-fluorophenylacetic acid (20 or 40 mM) or 2-fluoropropionic acid (40 or 80 mM) as the sole carbon and energy sources. Noble agar (15 g/L, wt/wt), bromothymol blue, and half of the DI water were combined, sterilized by autoclaving (15 min at 121°C), and cooled to 55°C in a water bath. The other MSB components, fluorinated compounds, and the other half of the DI water were combined, sterilized by passage through 0.2 µm PES membrane filters (VWR Avantor, Radnor, PA), and warmed to 55°C in the water bath. The two solutions were combined aseptically, mixed by gentle stirring on a magnetic stir plate, and then, 15 mL aliquots of the complete medium were poured into sterile 100 × 15 mm polystyrene Petri plates (VWR Avantor) (15 mL of medium per plate was optimal for observing color changes as the cells grew).

For plate studies, inocula were grown overnight in normal MSB with kanamycin (as described above) plus 25 mM of (*RS*)-mandelic acid or D-lactic acid for testing on α-fluorophenylacetic acid or 2-fluoropropionic acid plates, respectively. The cells were washed 2× with 1 vol of normal MSB before resuspension in the same media to OD600 = 0.1 or 0.2 (~1 or 2 × 10^8^ cells/mL). Cell suspension volumes of 1, 2, or 5 µL were spotted onto plates, which were then incubated at 28°C, and growth and color changes were observed over time.

### pH shift assays with purified DEF1

Substrate stock solutions (60 mM) were prepared by dissolving the fluorinated carboxylic acids in 4 mM Tris base (no pH adjustment), raising the pH to 9.5 with a 10 mM NaOH solution using a pH meter as above, and adjusting to the final volume with DI water. The assays were conducted in 96-well plates in 200 µL aliquots of 30 mM substrate in 2 mM Tris (pH 9.5) plus 0.2 mM *m*-cresol purple. The DEF1 solution (6 mg/mL) was added to experimental treatments at working concentrations of 1, 2, or 100 µg/mL, and the plates were incubated in the Tecan microplate reader at 30°C for 2–3 h without shaking. Absorbance changes were tracked at 585 nm. Controls with an equivalent volume of the DEF1 storage buffer (20 mM HEPES, 0.2 M NaCl, pH 7.5) in place of the DEF1 solutions were incubated in parallel.

### Encapsulating cells in alginate crosslinked with di- or tri-valent metal ions

A 2% (wt/vol) sodium alginate solution was prepared by completely dissolving 2 g of medium viscosity alginic acid sodium salt in 100 mL of DI water. The *P. putida* ATCC 12633 DEF1 + CLC^F^ psy strain was grown overnight to the stationary phase in LB with antibiotics as described above. Cells were harvested via centrifugation at 4,100 × *g* in tared 50 mL polypropylene conical centrifuge tubes (VWR Avantor), and the cell pellet was washed 1× with one volume of sterile normal saline (0.85% NaCl). After removing the supernatant, the wet weight of the cell pellet was determined, and the cells were resuspended in 1 mL of 0.85% NaCl per 0.1 g of wet cells. Crosslinking solutions of 0.2 M metal ions were prepared by dissolving CaCl_2_, BaCl_2_, SrCl_2_, LaCl_3_, AlCl_3_, or YCl_3_ in DI water.

Aliquots of the sodium alginate solution, with or without bromothymol blue added to 0.2 mM, were combined with the cells at a ratio (vol/vol) of 1:10 cell suspension:alginate solution and loaded into a syringe. A 22-gage needle was attached to the syringe, stirring of the crosslinking solution was started on a magnetic stir plate, and the alginate cells mixtures were dripped through the needle into the stirring crosslinking solutions from a height of 5 cm above the surface. When bromothymol blue was included in the encapsulation process, crosslinking was done only with a CaCl_2_ solution that had 0.2 mM bromothymol blue added, and the pH was adjusted to 7.5. When bromothymol blue was not included, encapsulation was done using each of the crosslinking solutions listed above. The formed beads were stirred for another 15 min and then stored in the crosslinking solutions at 4°C overnight.

### Assays with encapsulated cells

Following overnight storage, the formed beads were transferred into a clean beaker and rinsed with 3 × 1 vol of DI water. The entire batch was then decanted, blotted with paper towels to remove excess surface moisture, and weighed so that the mass of wet cell equivalents per gram of beads could be calculated. The beads were then transferred into 2 mM HEPES (pH 7.5) and gently stirred on a magnetic stir plate to equilibrate the beads with the buffer. The pH was monitored using a pH electrode and meter as above and adjusted with NaOH until it stabilized again at pH 7.5.

Stock solutions of α-fluorophenylacetic acid (80 mM) or 2-fluoropropionic acid (160 mM) were prepared in 2 mM HEPES, and the pH of each was adjusted to 7.5 with NaOH. Assay solutions were prepared by combining aliquots of the fluorinated substrate stock solutions with aliquots of 20 mM HEPES (pH 7.5), 1.5 mM bromothymol blue, and DI water to give working concentrations of 10-80 mM substrate, 0.05 mM bromothymol blue, and 2 mM HEPES at pH 7.5.

Duplicate assays for each treatment were started by blotting beads dry and weighing amounts that contained 0.0125 g or 0.025 g of wet cell equivalents for assays with α-fluorophenylacetic acid or 2-fluoropropionic acid, respectively, to give roughly equivalent rates of pH change with each substrate. The weighed beads were added to clean 50 mL conical centrifuge tubes, 10 mL of assay solution was added, and the tubes were laid horizontally in trays on a three-dimensional rocking platform set to cycle the solution and beads from one end of the tubes to the other ~30 × per min. Color change was monitored every 1.0–1.5 h (and again at 24 h) by removing solution aliquots from each assay tube, measuring absorbance at 615 nm with a Cary 60 spectrophotometer as above, and then returning each sample aliquot to its respective assay tube on the rocker. Free fluoride ion in each tube was measured every 1.5–2.0 h in 0.5 mL sample aliquots (not returned) using a fluoride electrode as described above.

## Data Availability

Data contributing to this study are provided here or are available in publicly available resources.

## SUPPLEMENTAL MATERIAL

Supplemental figures mbio.01798-25-s0001.docx Fig. S1 to S9.

ASM does not own the copyrights to Supplemental Material that may be linked to, or accessed through, an article. The authors have granted ASM a non-exclusive, world-wide license to publish the Supplemental Material files. Please contact the corresponding author directly for reuse.
