# Supplementary material for: Matrix-independent screening of defluorination in vitro and in vivo
Source: mBio. 2025 Aug 18;16(9):e01798-25. doi: 10.1128/mbio.01798-25 (PMC12421806; doi:10.1128/mbio.01798-25)
Supplement: Supplemental figures — Fig. S1 to S9. [file mbio.01798-25-s0001.docx]

1

2

3

4

5

6

7 Supplemental Figures

8

9

10 **Matrix-Independent Screening of Defluorination *in vitro* and *in vivo***

11

12

13

14

1. 2,3Anitha T. Simon, ^1,3^Anthony G. Dodge, ^3,4^Julie Bondy, ^3,4^Madeline R. O’Connor,
2. 2,3Alptekin Aksan, and ^1,2,3,4^Lawrence P. Wackett*

17

1. 1Department of Biochemistry, Molecular Biology and Biophysics, ^2^Department of
2. Mechanical Engineering, ^3^Biotechnology Institute, ^4^Program in Microbial Engineering,
3. University of Minnesota Twin Cities, USA

21


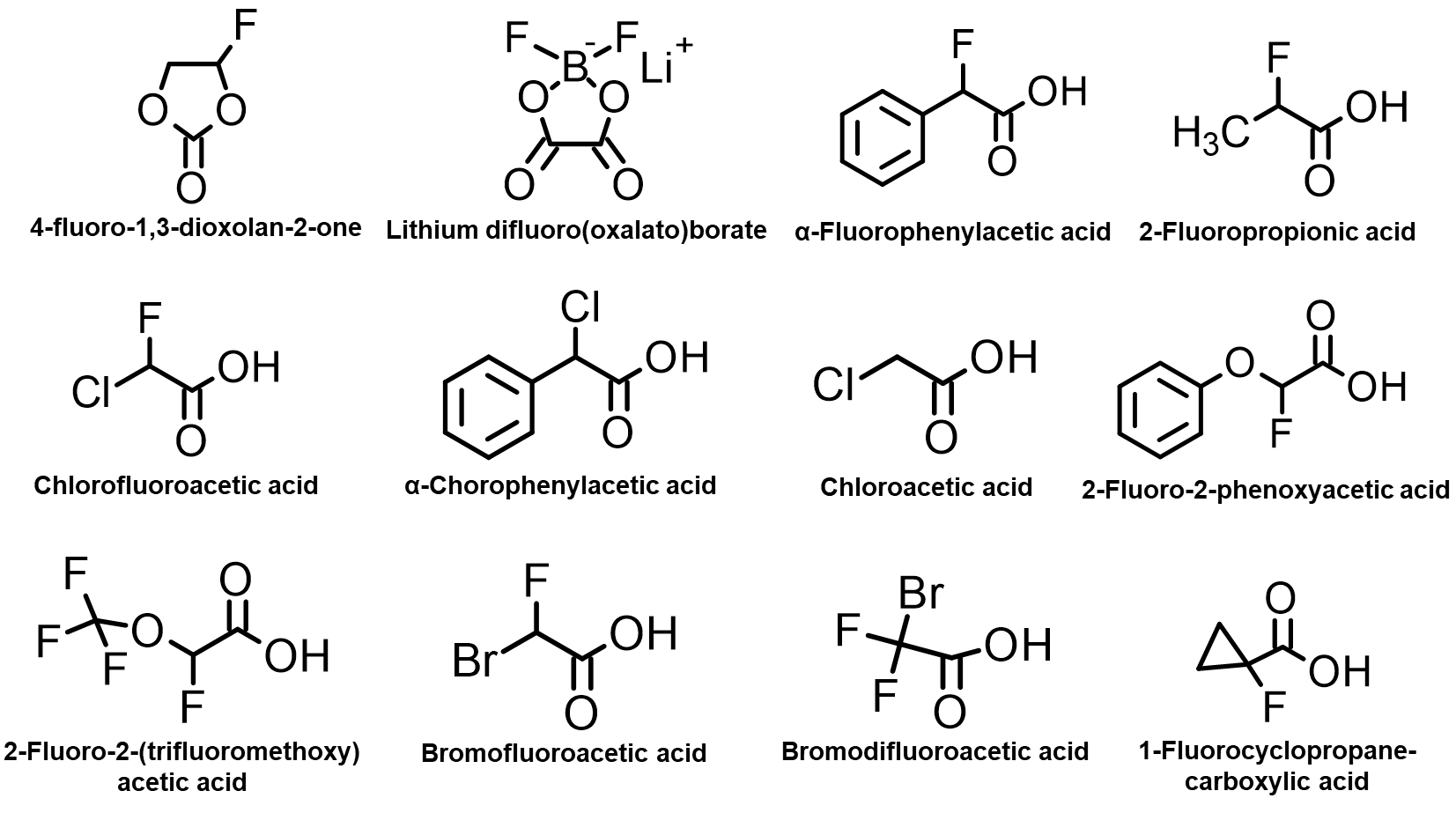


22

1. **Figure S1:** Structures and names of substrate chemicals used in experiments for
2. determining pH changes due to enzymatic or non-enzymatic dehalogenation reactions.
3. Structures were drawn using ChemSketch Freeware (ACD/Labs, Toronto, Ontario, Canada)


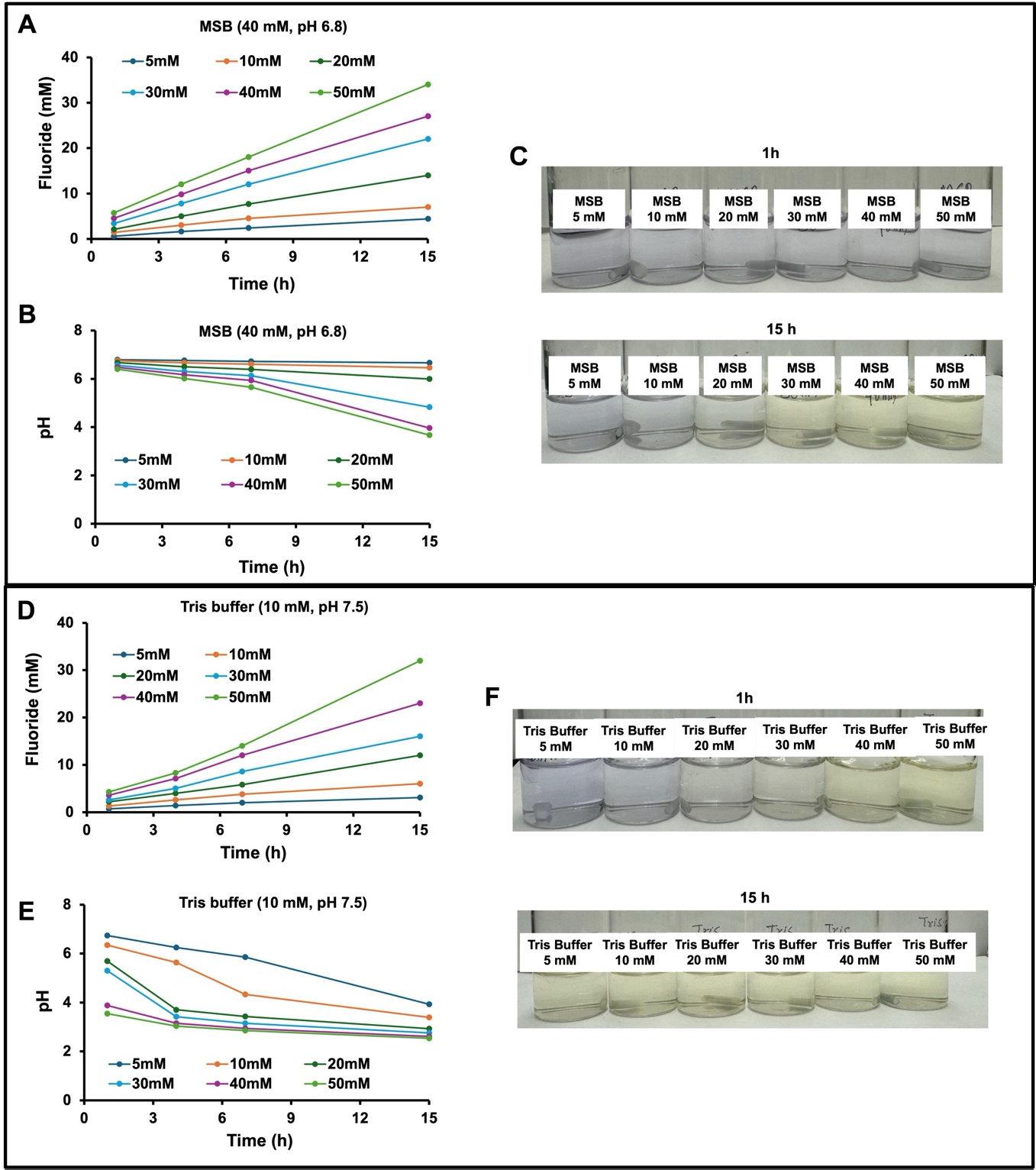


26

27

1. **Figure S2:** Fluoride release and pH change with different concentrations (5 – 50 mM) of 4-
2. fluoro-1,3-dioxalan-2-one in (A, B, C) normal MSB or (D, E, F) 10 mM Tris-HCl buffer (pH
3. 7.5). Defluorination was monitored in the presence of 10 µM bromocresol purple. (A, D) Plots of fluoride release or (B, E) pH vs time. (C, F) Photos of the assays taken after 1 or 15 h of incubation to show the visible color change.

33

35


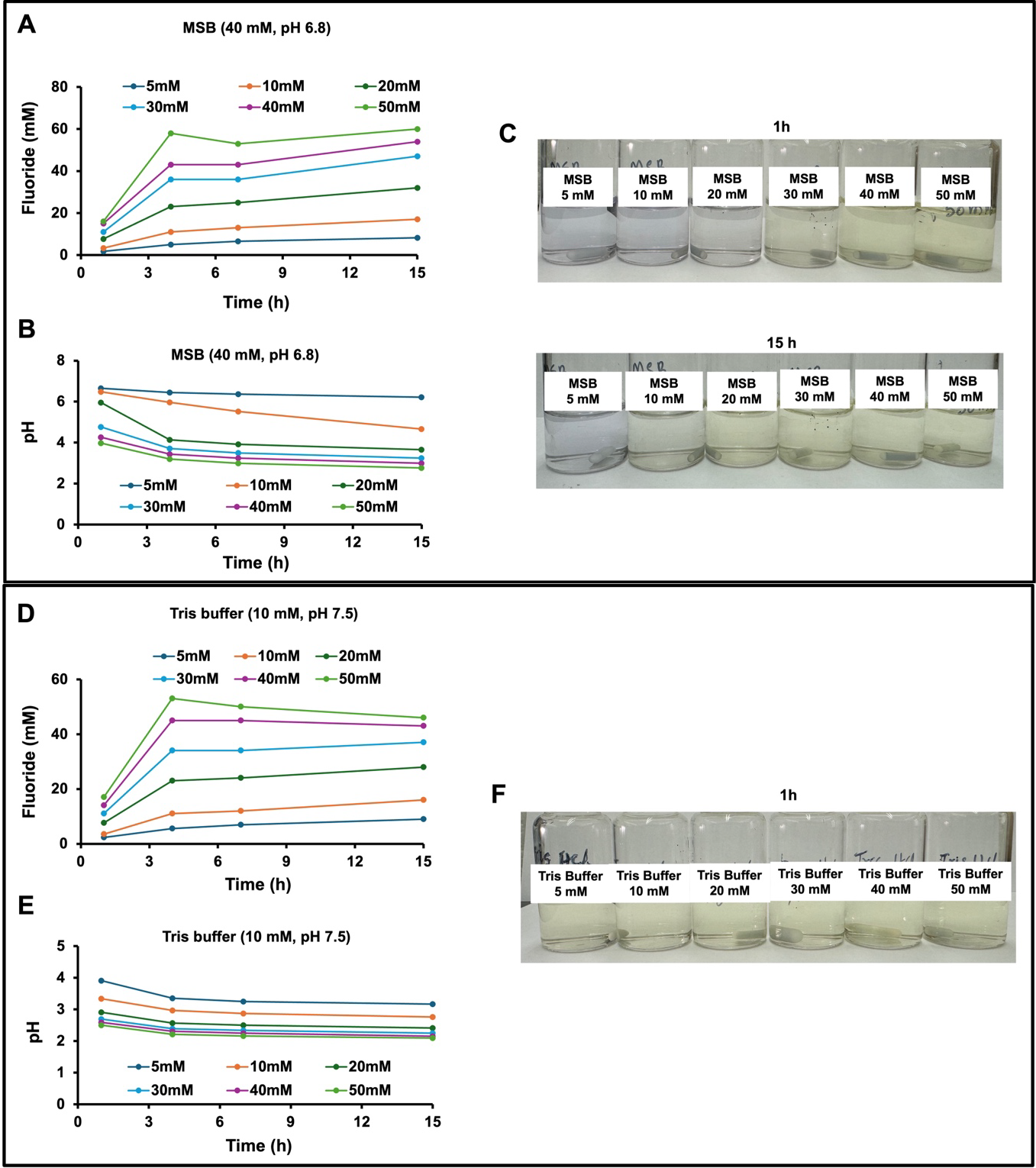
36

37

38

1. **Figure S3:** Fluoride release and pH change with different concentrations (5 – 50 mM) of
2. lithium-difluoro(oxalato) borate in (A, B, C) normal MSB or (D, E, F) 10 mM Tris-HCl buffer (pH 7.5). Defluorination was monitored in the presence of 10 µM bromocresol purple. (A, D) Plots of fluoride release or (B, E) pH vs time. (C, F) Photos of the assays taken after 1 or 15 h of incubation to show the visible color change.


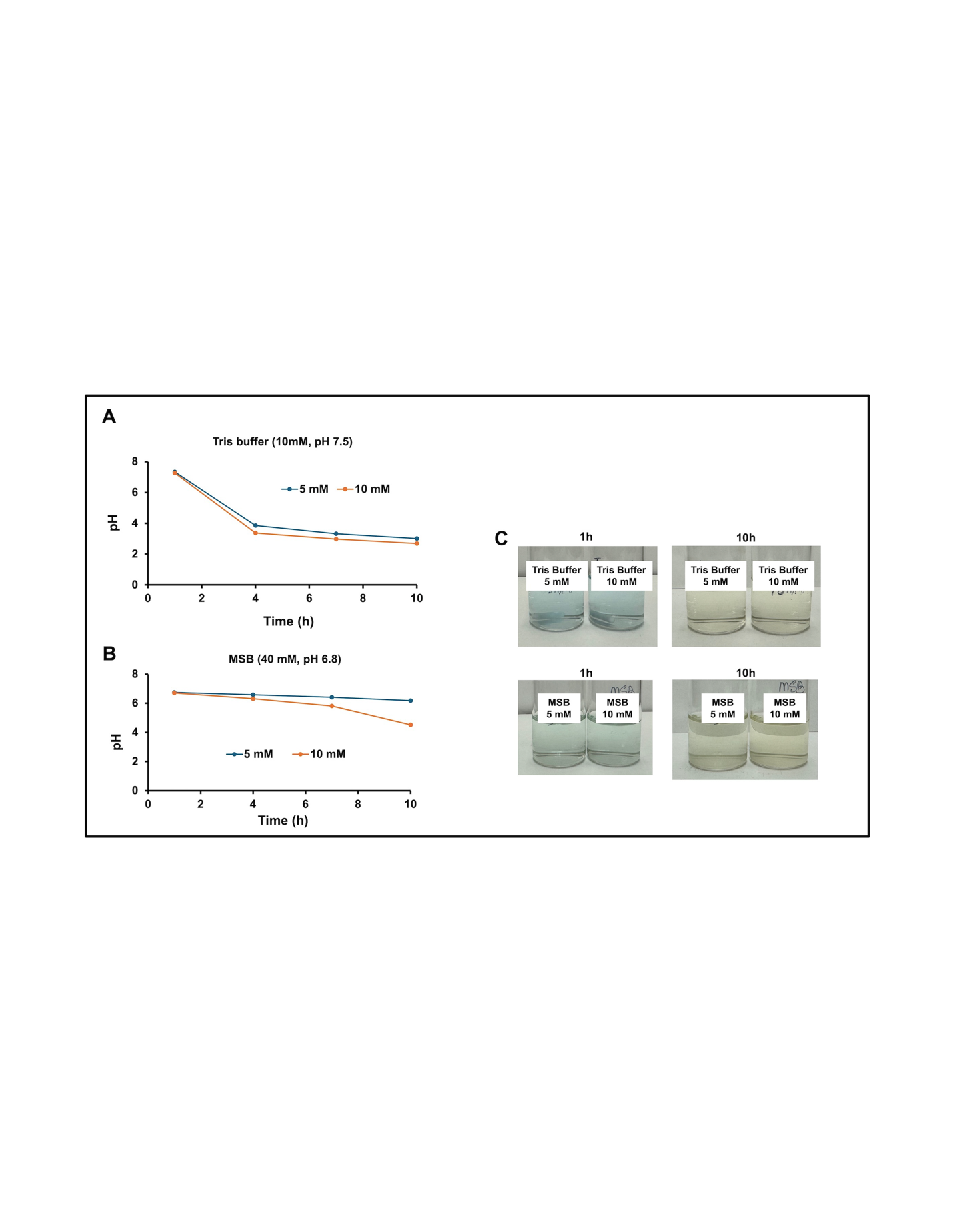


**Figure S4:** pH change with different concentrations of lithium-difluoro(oxalato) borate (5 –

1. 10 mM) in (A) Tris-HCl buffer (pH 7.5) or (B) normal MSB in the presence of 6 µM BTB.
2. (C) Photos of the assays taken after 1 or 10 h of incubation to show the visible color change.

55

56

57

58

59

60

61

62

63

64

65

66

67

68

69

71

72

73

74
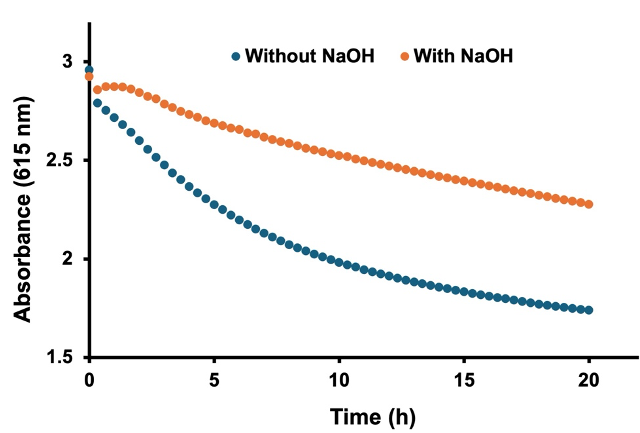


75

76

1. **Figure S5:** Mitigation of abiotic background acidification in weakly-buffered MSB medium
2. plus bromothymol blue (poised at pH 7.5) by adding NaOH to empty wells in the 96-well
3. assay plates. Rates of abiotic acidification were observed as a decrease in absorbance at 615
4. nm when the medium + indicator was incubated with shaking at 30 °C in a Tecan plate
5. reader/incubator (as described in the methods) with either 0.2 ml of (blue) water or (orange)
6. 5 M NaOH added to the empty wells.

83

85

86

87


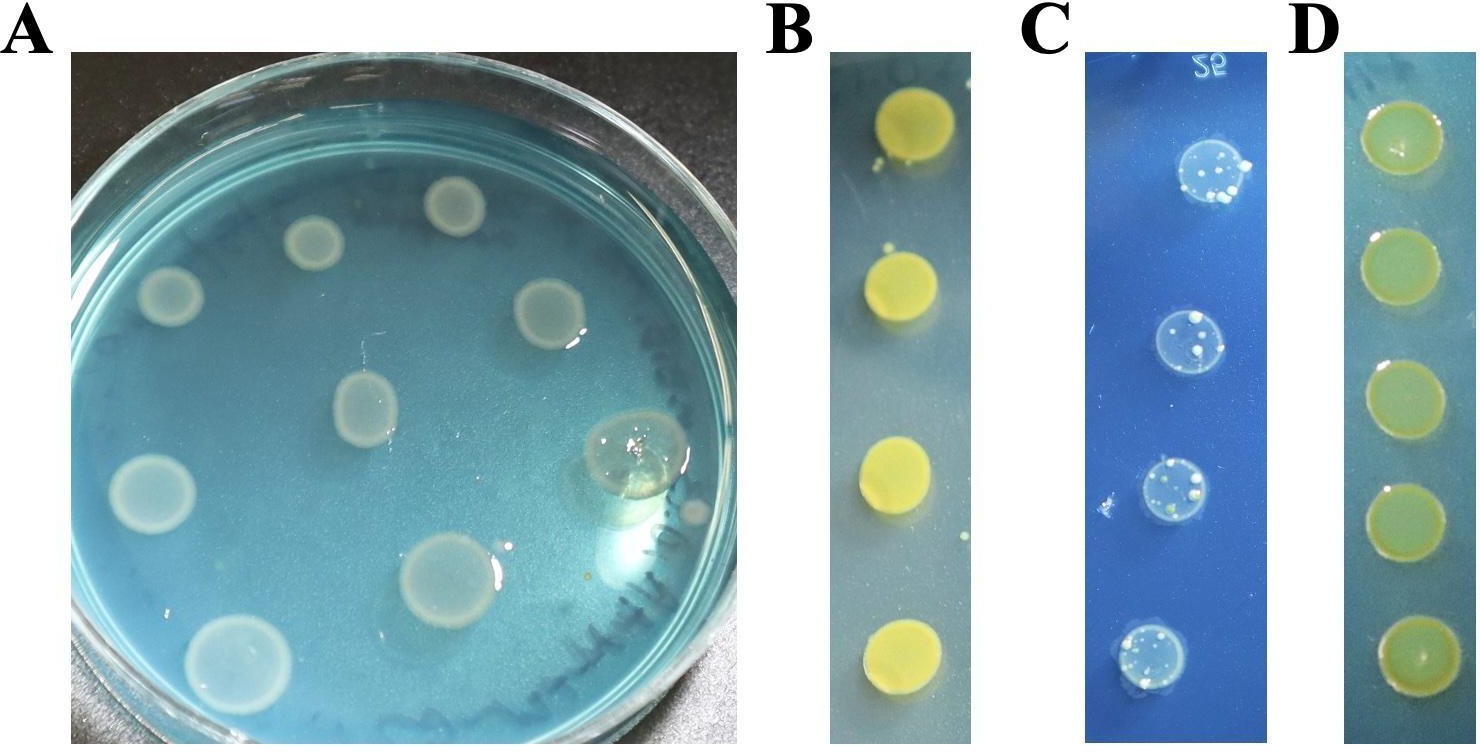


88

89

1. **Figure S6.** Determining optimum organofluorine concentrations, cell suspension turbidity,
2. and cell suspension volume for growth and defluorination by the adapted *P. putida* ATCC
3. 12633 + DEF1 strain on weakly-buffered MSB agar plates with bromothymol blue. (**A**)
4. Growth on plates with 20 mM α-fluorophenylacetic acid ten days after spotting 1 μL (top
5. row), 2 μL (middle row), or 5 μL (bottom row) drops of cell suspension at OD600 = 0.1 onto
6. the surface. (**B**) Growth on plates with 40 mM α-fluorophenylacetic acid six days after 2 μL
7. drops of cell suspension at OD600 = 0.2 were spotted onto the surface. (**C**) Growth on plates
8. with 40 mM 2-fluoropropionic acid nine days after spotting 2 μL drops of cell suspension at
9. OD600 = 0.2. (**D**) Growth on plates with 80 mM 2-fluoropropionic acid three days after
10. spotting 2 μL drops of cell suspension at OD600 = 0.2.

100


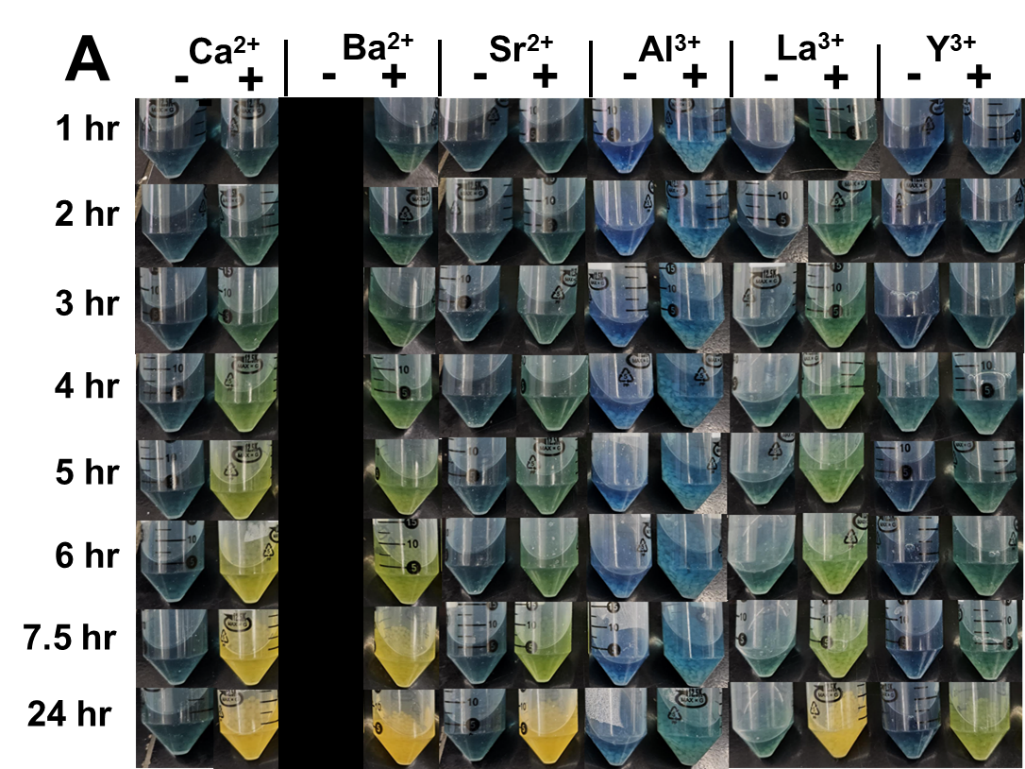
102

103

104

105

106

107

108

109

110

111

112

113

114


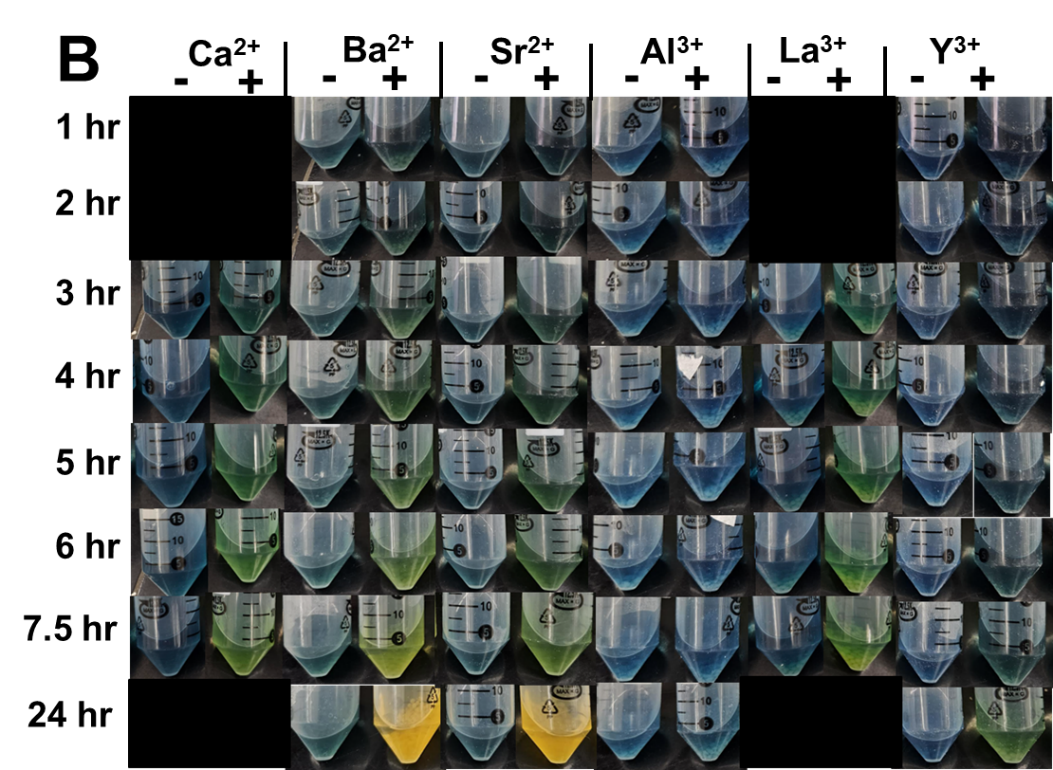
115

116

117

118

119

120

121

122

123

124

125

126

127

128

129

130

131

132

1. **Figure S7.** Composite photographs showing the complete time course of color change in
2. assays of fluorinated substrates incubated with DEF1 cells encapsulated in alginates
3. crosslinked with the indicated di- or trivalent metal ions. Assay solutions contained 2 mM
4. HEPES (pH 7.5) and 0.050 mM bromothymol blue. Negative controls (no substrate) are
5. indicated by “-”. Samples with (A) 80 mM 2-fluoropropionic acid or (B) 10 mM α-
6. fluorophenylacetic acid are indicated by “+”. Black space represents treatments that were
7. not included or were discarded due to error (for example, the incorrect assay solution was
8. used). The reactions shown use the same batches of encapsulated cells, but some are from
9. experiments conducted on different days.

142

144

145


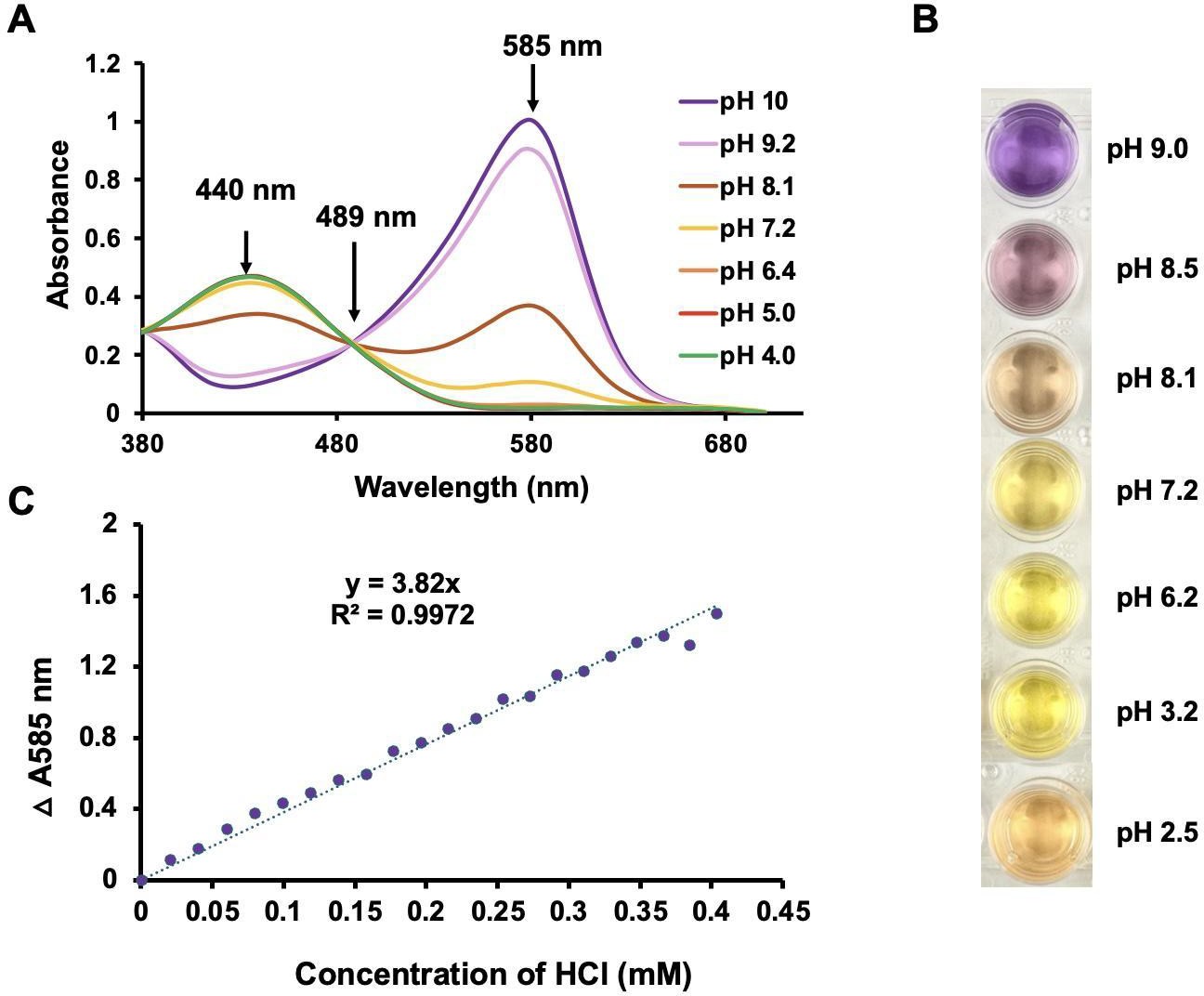


146

147

1. **Figure S8**: (A) Absorbance spectra of *m*-cresol purple (0.2 mM) in Tris-HCl buffer (2 mM)
2. recorded at pH range indicated. (B) Corresponding color change of *m*-cresol purple visually
3. observed. (C) Standard curve generated from a titration of *m*-cresol purple (0.2 mM) in 2 mM
4. tris-HCl (pH 9.5) with measured amounts of HCl.

152

153

154

155

156

157

158

159

160

161

162

164

165

166

167


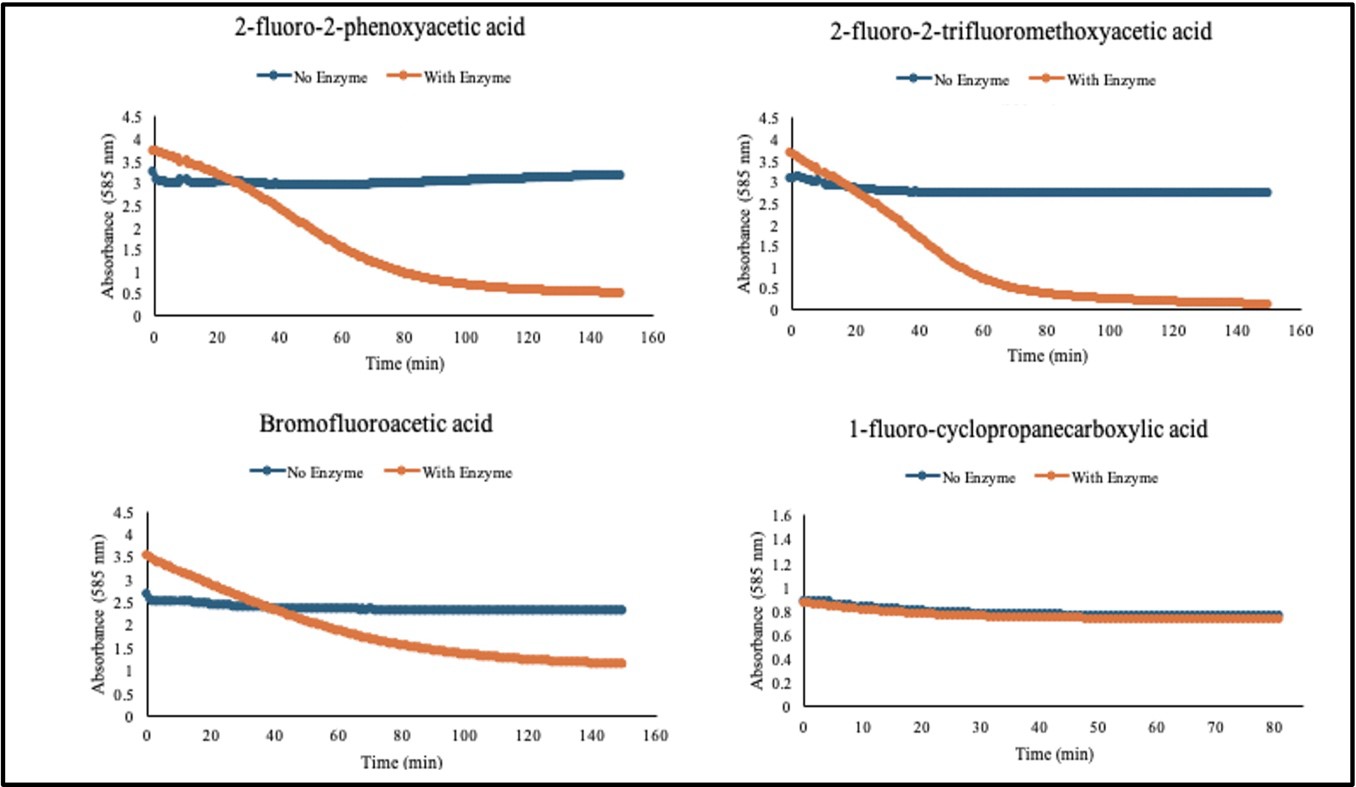
168

169

1. **Figure S9.** pH shift assays with purified DEF1 enzyme and previously untested fluorinated
2. compounds. Reactions were done in 2 mM Tris-HCl (pH 9.5) with 0.2 mM *m*-cresol purple
3. in 96-well plates in a Tecan plate reader as described in the Methods section.

173
